# Exploring the environmental determinants of food choice among Haudenosaunee female youth

**DOI:** 10.1186/s12889-022-13434-z

**Published:** 2022-06-09

**Authors:** Rebecca Hanemaayer, Hannah Tait Neufeld, Kim Anderson, Jess Haines, Kelly Gordon, Kitty R. Lynn Lickers, Adrianne Xavier, Laura Peach, Mwalu Peeters

**Affiliations:** 1 Department of Family Relations and Applied Nutrition, The University of Guelph, Guelph, ON N1G 2W1 Canada; 2 School of Public Health Sciences, The University of Waterloo, Waterloo, ON N2L 3G1 Canada; 3 Six Nations Health Services, Ohsweken, ON N0A 1M0 Canada; 4 Indigenous Studies Program, Department of Anthropology, McMaster University, Hamilton, ON L8S 4L8 Canada

**Keywords:** Indigenous, Youth, Food literacy, Nutrition, Food environments, Social determinants

## Abstract

**Background:**

Research on Indigenous food literacy within Canada has been focused on northern and remote communities despite the fact there are considerable and unique barriers to food access, availability, and utilization in southern Indigenous communities. Food insecurity is also a prevalent issue among Indigenous Peoples living in these more populous regions. Study objectives included investigating the determinants of food choice among youth, along with perceived opportunities that would improve food environments individually and at the community level.

**Methods:**

This community-based study used Photovoice to explore the perceptions and experiences of traditional foods and the determinants of food choice among youth in the community of Six Nations of the Grand River. Participants took photos of their local food environments, including where foods were acquired, consumed, prepared, or shared, and participated in semi-structured interviews to share the stories behind these images. Thematic analysis was used to identify patterns in participants’ photos and interview content.

**Results:**

Environmental factors were found to influence participants’ traditional and everyday food choices. Built, social, economic and ecological environments were described by the youth as distinct yet inter-related determinants that converge to influence individual food choice. Built environments had a notable impact on food choice, most notably at home and in school settings. Home and family were found to be facilitators of meal consistency and healthy food choices across participants. The social environment including participants’ relationships with their peers and community friends was often a barrier to healthy food choices. Eating at fast food outlets was a common social activity. The economic environment included cost deterrents associated with food choices and regular meals. The ecological environment was less of an influence and impacted the seasonal consumption of traditional and other locally harvested foods.

**Conclusions:**

Overall, the study findings have generated important knowledge regarding food environments and literacy and serves as a unique example of how to explore the traditional and everyday food experiences of Indigenous youth. Recommendations will inform the development of new as well as existing initiatives and resources to enhance the holistic wellbeing of youth and the broader community.

## Introduction

Relationship with the land, developed and maintained through reciprocal practices, is an essential component of the health and wellness of Indigenous Peoples [1, 2]. Traditional^1^ foods and foodways are an extension of this relationship, which uphold physical, mental, spiritual and cultural wellbeing [1, 3–6]. As a part of larger, multifaceted traditional food systems, which encompass the sociocultural meanings, processing techniques, composition, and nutritional consequences for Indigenous Peoples using these foods [3], traditional foods are unique to Indigenous communities with respect to local ecosystems and biodiversity [7, 8]. While Indigenous groups continue to recognize the important contributions traditional foods can make toward holistic wellbeing, traditional food consumption among Indigenous Peoples within Canada has gradually declined over time [6, 9–11] with changing food environments. Though most Indigenous adults report consuming traditional foods, the majority does not habitually engage in traditional food practices [12–15] despite a collective desire to consume more of these foods [3, 12, 16]. According to the First Nations Food and Environment Study (FNFES), traditional food consumption was found to be the highest in more northern and western ecozones compared to the Prairies, Mixedwood Plains, and Atlantic Maritimes [12]. The Mixedwood Plains covers the most southern territories within Canada. Traditional food consumption is less frequent in these southern regions compared to those that are more rural and remote [13] with 29.5% of First Nations adults reporting regularly accessing wild meats and only 18.1% harvesting plants from the local environment [13].

This gradual westernization of Indigenous diets is a global phenomenon frequently referred to as the nutrition transition [6, 17]. These processes of colonization have led to high levels of food insecurity and the degradation of food environments by depriving Indigenous people of land, culture, language, and relationships [1, 18]. While Indigenous Peoples have been disconnected from their land, food, and medicines through these direct and indirect processes of environmental dispossession, political and legal authority over their Nations and Territories has been interrupted by the imposition of settler-colonial state structures, resulting in detrimental impacts on physical and social environments [19]. Colonization has disconnected Indigenous Peoples from culture, family, and community [18]. Most notably, the on-going legacy of the residential school system has undermined the fabric of Indigenous communities and disrupted intergenerational sharing and dismantled relationships with local food environments [20]. Children and youth attending residential schools were denied access to traditional foods and food procurement activities, and served poor-quality meals that lacked variety, taste, and nutritional value. Since relationships with food are socialized, attending residential schools contributed to a disconnection from culture, food, and the land [1, 18, 20].

The majority of research that has taken place investigating food consumption behaviours among Indigenous Peoples within Canada has utilized primarily quantitative methods. Research investigating preferences and attitudes toward food is also lacking, particularly among Indigenous youth. This is problematic as almost half of Indigenous Peoples within Canada are under 25 years of age [21]. In addition, the majority of studies investigating dietary patterns have found that traditional food consumption increases with age [12, 22, 23]. At the same time, Indigenous youth consume the highest quantity of nutrient deficient ultra-processed foods [14, 23, 24]. These dietary trends and nutritional risks factors are based on limited evidence as most existing studies have taken place with adults. Little is known about the determinants of food choice influencing the health and wellness of Indigenous youth within Canada [10]. The participation of youth in community engaged food literacy is necessary to revitalize and sustain food environments and practices for generations to come [1, 7, 25–28].

Most literature on Indigenous food literacy within Canada has been focused on northern and remote communities [23, 24, 29] despite the fact there are considerable and unique barriers that reduce food access, availability, and utilization in southern and urban Indigenous communities. Food insecurity is also a prevalent issue among Indigenous Peoples living in southern and urban regions [3, 13, 30]. Health care practitioners have identified a lack of resources within these contexts across all life stage groups [3, 17] and limited engagement of Indigenous Peoples living in these more populous regions. To explore the multi-dimensional determinants of food choice among Indigenous Peoples across life stages and geographical contexts, community engaged research is more methodologically suited.

This study was designed to address these identified gaps in the literature, while building on existing community interests following several years of consultation and grant preparation to inform local programing within the Haudenosaunee community of Six Nations of the Grand River. The overall aim of the study was to explore the perceptions of and experiences with traditional foods among youth in the community, as well as the determinants of food choice. Study objectives included investigating: (a) how the youth understand, define, and value traditional foods; (b) traditional food experiences of youth, including growing, acquiring, preparing, consuming, and learning; (c) the determinants of food choice among youth, including perceived facilitators and barriers to their availability, access, and use; (d) perceived opportunities that would improve food environments individually and at the community level. Findings reporting on objectives (a) and (b) have been published elsewhere [31]. This paper concentrates on objectives (c) and (d).

## Community setting and study background

Six Nations of the Grand River Territory is the largest First Nations community within Canada, located in present day southernwestern Ontario. Made up of a total of approximately 26,000 band members, just under half of these members live on-reserve [32]. In recent years there has been a growing interest in reconnecting with Haudenosaunee foodways [4]. Haudenosaunee translates as the “People of the Longhouse”, often referred to as Iroquois. The Six Nations were originally comprised of Mohawk, Cayuga, Oneida, Seneca and Onondaga Nations, with the eventual addition of the Tuscarora Nation [4]. In 2012, the community of Six Nations participated in the FNFES, at which time multiple barriers to accessing traditional foods were identified, including as access to traditional knowledge. Overall, 73% of participants in this study identified that they would like to consume more traditional foods [12]. Indigenous families in nearby communities and cities also participated in the Southwest Aboriginal Health Access Centre’s (SOAHAC) Food Choice Study [16]. Results indicated that 76% of urban-based families and 52% reserve-based families were interested in consuming more traditional foods.

In the years preceding this study, several initiatives in the Six Nations community began building momentum and interest around traditional food systems. *Our Sustenance* was a program that was founded in 2011 in response to community desire for accessible fresh foods, and organizes activities that included a farmer’s market, community garden, and weekly food basket program [33]. In 2015, Six Nations Health Services and the local news publication, *Two Row Times,* introduced the Healthy Roots Challenge, a 90-day community nutrition intervention, which involved consuming only pre-contact foods^2^ and participating in daily physical activity. After a successful launch, this initiative became an annual event and grew to advance local food sovereignty initiatives by promoting interconnectedness and enhancing traditional food and knowledge access [4]. In 2016 a community-based dietary intervention study was initiated in partnership with McMaster University to measure cardio-metabolic factors of participants. Improvements in blood glucose control, weight, and ancestral food knowledge were reported [4, 34]. Events organized to engage the wider community in reconnecting to activities, such as cooking and gardening workshops, and a Haudenosaunee Food Guide were also recently developed to raise awareness and promote locally harvested foods from the skies, meadow, garden, woods and water [4].

The success of these activities fostered a growing community interest in reconnecting to traditional foodways as a means of enhancing wellbeing. Six Nations Health Services staff were interested in exploring further opportunities to respond to on-going community interests. The overall idea and vision for the present study arose from discussions with health promotions staff, community members and academic partners. Beyond developing the initial idea and vision for the study, this partnership continues to champion the ongoing development of this research project through community-based participatory research (CBPR). Reflecting these relationships, the authors of this article include community (KG, KRL, AX) and academic (HTN, KA, JH, AX,) researchers and students (RH, LP, MP) of varying roles and responsibilities within this study and the wider research initiative within which it is situated.

## Methodology

Indigenous methodologies represent alternative ways of thinking about research processes [35]. They tend to encompass more fluid and dynamic approaches to support ethical and respectful relationships towards research that thoughtfully integrate Indigenous worldviews and knowledge [35]. Research should also be adapted to consider the unique context and needs of each cultural group and community [36]. From an epistemological standpoint, Indigenous methodologies recognize that there are multiple realities and that truth exists in subjectivity [36]. As such, knowledge is gathered and generated in a way that is subjective in order to explore and capture the complexity and nuance of human experiences [17, 36].

This study was nested in a larger research project that is investigating the impacts of place and space on urban and reserve-based food environments within Haudenosaunee territory. From the start, a CBPR approach was utilized, due to its decolonizing framework and strengths-based approach imperative to Indigenous research contexts [37, 38]. The main goals of CBPR include: minimizing researcher-participant power dynamics, building community capacity, fostering trusting relationships, and developing a community sense of ownership of the research project [37–39]. The research was therefore designed in collaboration to support the interests and vision of Six Nations Health Services staff and leadership, as well as provide opportunity for capacity building. A local research assistant was hired to work with the lead author, and a Community Advisory Group established that met with the research team and Health Services staff to guide research implementation and the on-going dissemination of results within the wider community.

Photovoice shares many core principles with CBPR, such as minimizing researcher-participant power dynamics by providing participants with creative autonomy [36, 37, 40–42]. Photovoice is a flexible, arts-based methodology where participants take photographs to capture their views and experiences related to the research questions. As such, it effectively attends to the importance of subjectivity within Indigenous worldviews and understanding [36]. Further, Photovoice projects strengthen participant capacity [36, 42, 43], support relationship building [36], and centre the exploration of topics and issues of interest to community members [36, 37, 44]. As a methodology, Photovoice has also been identified as appropriate and ethically sound to use in research with Indigenous youth [41, 42, 45, 46].

### Data collection

Upon receiving ethical approval from Six Nations Ethics Committee and the University of Guelph’s Research Ethics Board (REB #: 17-03-034), outreach and recruitment began. Multiple purposive sampling strategies were utilized, including recruitment posters, information sessions with community youth groups, social media advertising, and word-of-mouth. From February to July 2019, a total of five youth expressed interest in the study and were recruited to participate. Three participants took their photos during the winter months, and two participants during the summer. A brief description of the participants is provided in Table 1. All self-identified as female, and were between the ages of 15-22. The youth attended either high school (*n* = 4) or college (*n* = 1) at the time of their participation.

**Table 1:** Participant characteristics

Participant Pseudonym	Gender	Age	Educational Background
Maria	Female	15	High school student
Grace	Female	15	High school student
Melody	Female	15	High school student
Georgia	Female	16	High school student
Margaret	Female	22	College student

Prior to beginning data collection, the lead author and community research assistant met with each participant to provide an overview of the research project, obtain informed consent, and answer any questions. Youth were trained on how to use the digital cameras provided to them and instructed to take an unlimited number of photos that captured their food environments or everyday food choices and experiences, including consuming, acquiring, growing, preparing, or sharing foods.

The lead author collaborated with the research team and Community Advisory group to develop a semi-structured interview guide that comprised open-ended questions about participant’s personal and family food choices, as well as their understandings and experiences with traditional foods. The interview guide was piloted before participant interviews began. Once participants had completed their photography assignment, the community research assistant arranged face-to-face participant interviews at a date and location of the youth’s choosing. Each youth was asked to select 8-10 photos to discuss during the interview. These discussions were audio-recorded with permission, and lasted between 64 and 79 minutes in duration. As an honorarium for their participation, youth were given a $50 Visa gift card and a framed copy of their favourite photo.

### Data analysis

Interviews were transcribed verbatim once data collection was complete. Member checking was then sought individually from each participant to ensure any potentially identifying or other sensitive details were removed from the transcripts. NVivo 12 qualitative research analysis software was then utilized by the first author to manage and analyze the data collected, including photos and interview transcripts. As participants analyzed and assigned meanings to their photos during interviews, general thematic categories were allocated to the photos to identify types and sources of foods captured, how and with whom food was consumed, and places of food consumption. The collected photos were organized and saved with their corresponding interview transcripts so that they could be analyzed for themes concurrently within NVivo. Once these codes were assigned to each photo, overarching and recurring themes related to the content of the photos were identified. Thematic analysis was guided by Braun & Clarke’s [45, 47] six-stage iterative process of analyzing and describing data, and relating identified themes to the research objectives, with support from academic supervisors and later members of the wider research team, including research partners along with members of the Community Advisory group.

## Results

**Fig. 1:**
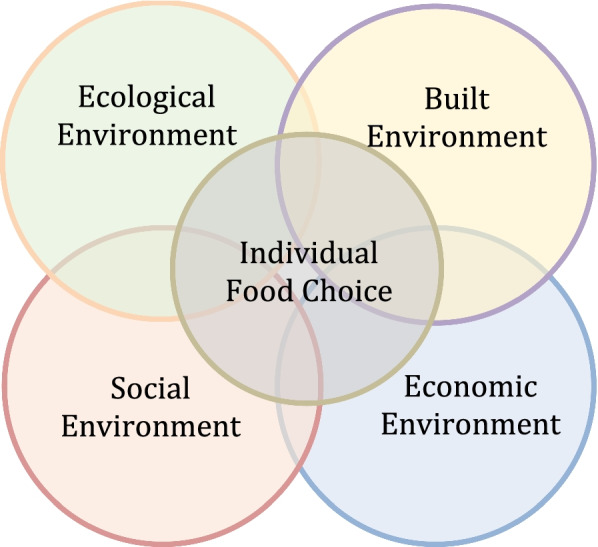
Environmental determinants of food choice

The results presented are framed by adapting environmental determinant categories identified in previous research [48, 49] to reflect their distinct influences identified by the youth in this study. Specifically, this research acknowledges the social (e.g., family, peer influences), physical (e.g., school, restaurants), and economic environments (e.g., income, socioeconomic status) identified by Raine [48] and Larson and Story [49], and expands these constructs to accurately reflect participant observations around the natural environment. As environmental determinants refer to broader, contextual factors that impact individual eating behaviours [48], the determinants found in this study are encompassed by four inter-related environments: built, social, economic, and ecological (Fig. 1). The built environment, like the physical environment mentioned in previous research [48, 49], refers to the human-made places participants spent much of their time engaging in food behaviours and practices, such as schools and restaurants. Social environments encompass the influences of relationships with family members, peers, and community. Similarly, economic factors, either individually or within households, were also identified. Finally, and unique to this study, the impacts of ecological or natural environments on participant food choice is also represented in the model.

### Built environments

During their interviews, participants described how the foods they consumed, prepared, and purchased changed based on location, or the places where they spent most of their time. The places with the most influence on their food behaviours were home and school. Home environments were talked about as a central influence on participants’ food choices. All of the youth lived with family members and most were solely reliant on them to purchase food and prepare meals. The one exception was Margaret, who shared that acquiring and preparing foods was often a collaborative effort*.* She explained, *“I help out with groceries and stuff when I can… me and my mom usually, split it ‘cause we’re the only ones working at home.”*

Morning and evening meals were typically consumed at home. Participants described preparing their own breakfast, although only one participant reported habitually eating a morning meal. In contrast, dinner was the most consistently consumed meal, with the participants’ parents primarily responsible for its preparation. All participants reported that evening meals were typically home-cooked, with occasional takeout or fast food alternatives. Melody described how dinner settings varied: *“When I stay at my dad’s we go out all the time… so Wednesdays and Thursdays I’m with him, so we usually eat somewhere… and then at home Monday nights my mom always has a home-cooked meal.”*

Participants also expressed how these home-cooked dinners were sources of a variety of nutritious foods compared to the meals they prepared for themselves or consumed elsewhere. Georgia explained that, *“if I was going out, I’d get hamburgers and stuff… but when I’m at home* [I] *have meat and potatoes and stuff like that… stuff* [that is] *better for you.”* Maria talked about how dinner was often a source of variety in her diet because of her parents’ responsibility for food preparation: *“The only time everything really changes up is for dinner. It’s ‘cause dinner’s not up to me… it’s up to my mom… But lunch and breakfast, those are my choices, so it kinda just stays the same.”*

**Fig. 2:**
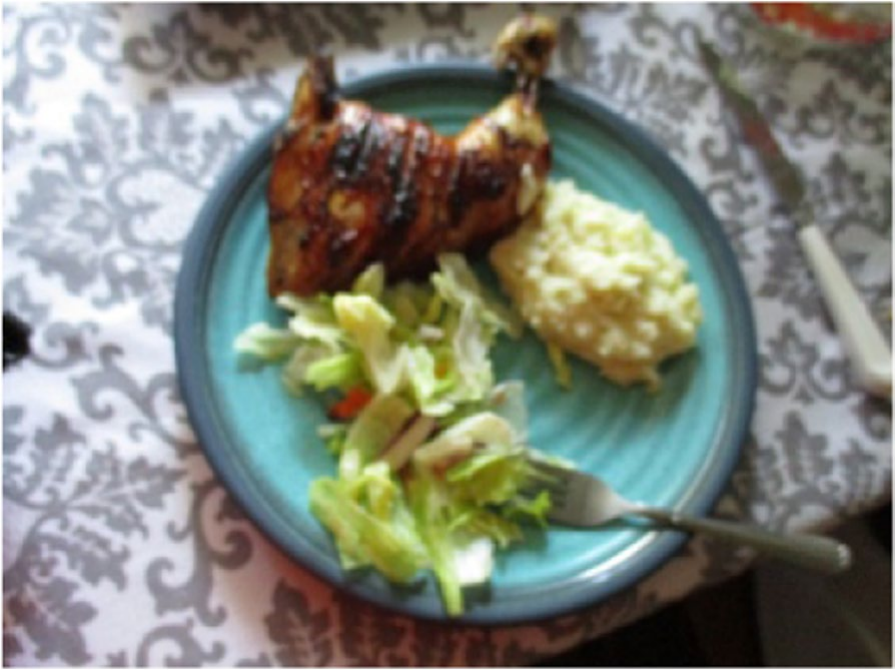
Homemade family dinner (Georgia, 16)

Though all participants talked about home-cooked dinners, not everyone captured photos of their evening meals. Georgia was one of the two participants (sisters from the same household) who captured photos of dinners consumed at home. She explained her family only recently began having more homemade meals due to shifting responsibilities when talking about her photo (Fig. 2). She elaborated, “*well [*my mom*] just started making more food… she didn’t really cook that much ‘cause she’s like ‘you guys are all old enough to cook for yourselves so you cook’.”*

**Fig. 3:**
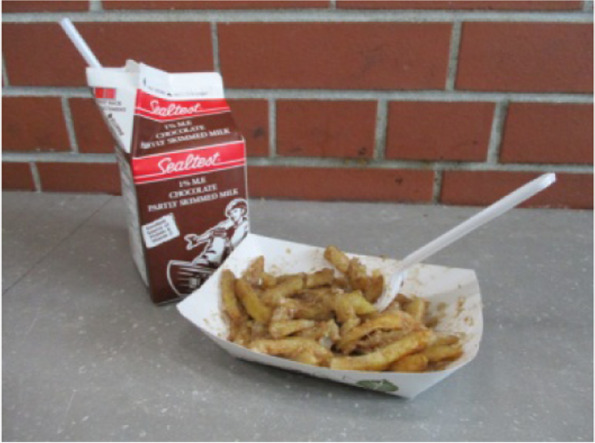
Purchased cafeteria lunch (Melody, 15)

The school environment also influenced the food choices of participants. All of the participants were students. However, only the three students who were involved in the study during their academic year captured photos of their food environments at school during the winter months. Despite this, everyone talked about foods they purchased and ate at school. Ultra-processed foods were the mainstay of their school diets. Participants acquired foods from their cafeteria or nearby coffee shops, pizza “joints”, and convenience stores. Of the ten photos Melody captured, six were of meals she had purchased in the cafeteria (Fig. 3). She acknowledged the foods she had at school were less nutritious, stating, *“at school I probably eat unhealthy… a little more.”* She felt the foods available in the cafeteria were a significant influence on the quality of students’ lunches generally, theorizing that: *“if schools had more healthier options, then kids would be healthier.”* Maria was the only participant who talked about packing a lunch for school, despite her preference for eating out*.* As she explained, *“I used to pack a lunch almost every day in grade 9, but now… when I do pack a lunch it’s more if I used up all the money… or I just need to save it.”*

**Fig. 4:**
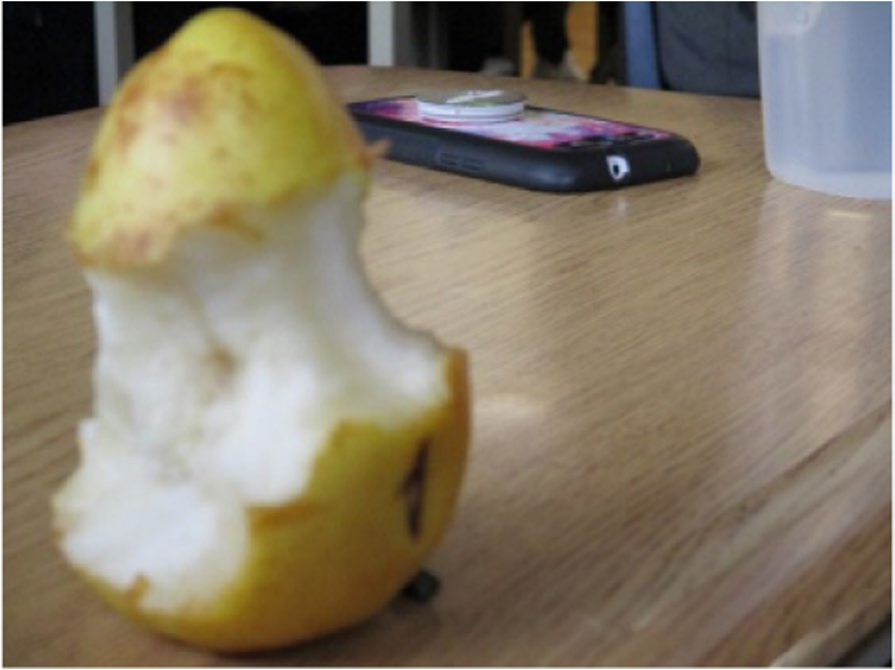
Food from school program (Grace, 15)

Participants also reported their meal patterns at school were more variable than meal patterns in other settings. Margaret, a college student, described eating breakfast more regularly when attending school, but her overall intake was inconsistent on school days. She explained, *“I’ll eat lunch when I’m at work, but if I’m at school I won’t eat lunch… I won’t eat until dinner time.”* Participants attending high school reported occasionally missing lunch due to a lack of money or time. Several accessed the Student Nutrition Program when they did not have money to buy food, forgot to pack a lunch, or did not have time to eat breakfast. Grace captured a photo (Fig. 4) to illustrate a time when she and her friends accessed the program and described her experience:

*“We all didn’t have breakfast that day, so we’re like, ‘man we’re kinda hungry’… and so we’re like ‘can we go get a fruit cause we didn’t eat breakfast today?’ and* [the teacher said] *‘yeah sure whatever’. So after he let us go down to the office and get fruit.”*

Three women spoke about how they had enjoyed learning food skills through high school foods classes. These same three participants also described a desire to enhance their food preparation abilities. Margaret talked about dishes she was interested in learning how to make*,* such as *“authentic Mexican food…[and] jambalaya.”* Motivation to improve her food skills and gain more independence was to eventually *“move out of the house.”* Melody shared her thoughts on the broader value of food skills programs for young people: *“I feel like a good program… would be to teach kids how to cook… ‘cause I eat fast food like three times a week probably… at my school. So I just feel like if you introduce kids to cooking young then that’s what they grow up doing.”*

### Social environments

**Fig. 5:**
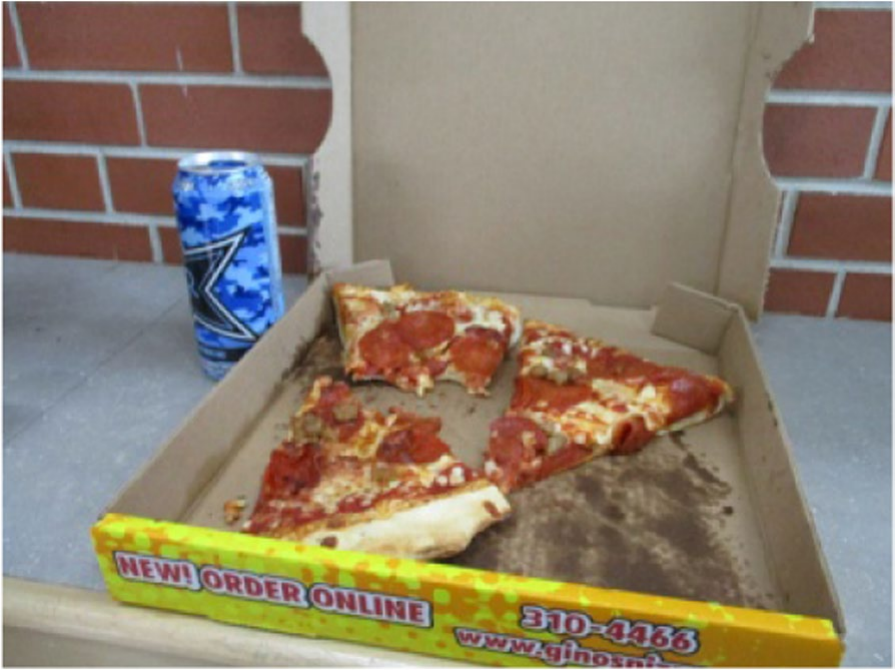
Shared meal with school friends (Melody, 15)

From the participants’ photos and interviews, it was apparent food was an integral part of socializing with friends and family. The majority of photos shared captured contexts where food was consumed or shared with others. Photos taken by three female youth during the school year clearly illustrated the importance of these opportunities to socialize during lunch breaks and after school. Melody talked about a weekly lunch tradition she kept with her school friends as she described the photo in Fig. 5, *“everyone will pitch in to get a pizza… and then we’ll just share slices.”*

**Fig. 6:**
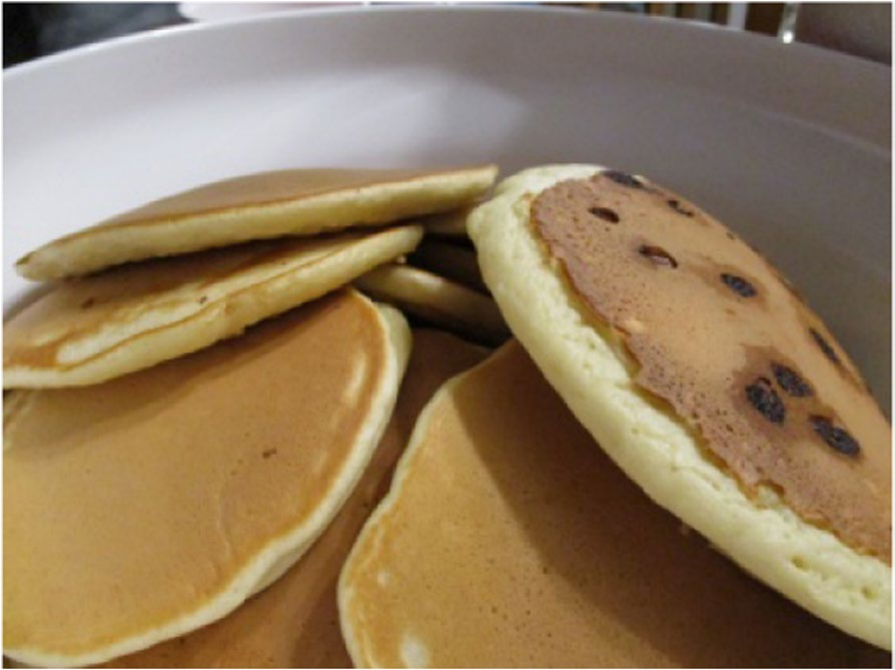
Family food traditions (Grace, 15)

Participants told stories about sharing food with their families. All spoke about having home-cooked evening meals prepared by a family member, however, their unique social environments influenced whether or not these meals were a social event. Margaret explained how family dynamics could impact mealtimes together, explaining that *“usually, everyone just does their own thing…I just make food for me and [*my son*]. If they wanna eat it then they can… but everybody fights so… it’s not pleasant.”* Several youth captured photos of meals shared with extended family. Maria talked about these spontaneous meals, recalling, *“on the weekends sometimes…my aunt…gets a* pizza [and] *hit up the other fam and they’re all over and we’re all eating pizza. And it’s not just pizza… this weekend we just had beef stew… we had like two pots of it for everyone.”* Grace described her extended family’s food traditions as well. In response to her photo (Fig. 6), she explained: “*it’s* [Pancake Tuesday] *like something annually that… my family tends to enjoy… so* [my papa]*'s got a big table so usually we’ll all sit at the table.”* Though Margaret and Georgia reported limited connection to their extended family, they both spoke about seasonal family traditions that involved food. Margaret said that *“around the holidays my brother’s family comes over a lot and we eat dinner every Sunday during football season. It’s like the same meal every time.”*

Several youth also discussed how their friends and family influenced their food preferences and choices. Maria explained, “[my friends] *could have something I’ve never had before. They say they like it… I can ask to try it or if they offer it I’m always like ‘sure.’”* She also gave examples of how family members had influenced her food preferences, including *“my aunt had a green tea, two sugar. I kind of was like ‘oh this stuff’s good’ so I’d get it too… and I think my dad got me into coffee”.* Melody shared that the types of foods she ate at school were influenced by her friends since, *“I usually have lunch with… a lot of people so… my friends who like to go outside that’s where we’ll go out… like my two closest friends, we always just go to the* [cafeteria] *and we stay inside. Or we go to our friend’s foods class and eat in there.”* As a young mother, Margaret talked about how having a child influenced her willingness to try new foods*.* She said, *“I’ve been trying to* [eat new foods] *lately so I can have more recipes for* [my son] *just ‘cause I’m a parent now… I want to make sure he’s eating healthy…‘cause I want him to have better eating habits than I did when I was a kid.”*

Margaret was the only participant that discussed her involvement preparing meals at home with her family. Of the forty-six photos taken by participants, only six photos captured meals that had been independently prepared; two depicted meals that participants assisted preparing. The majority of meals that were photographed captured used ultra-processed foods or items requiring minimal preparation. For example, Melody described simple meals such as, *“prepping small stuff in the morning like sliced oranges and bananas and stuff like that.”* Similarly, Maria said, *“now that we’re older it’s more we do our own thing, we fend for ourselves basically, so we gotta make our own breakfast.”*

**Fig. 7:**
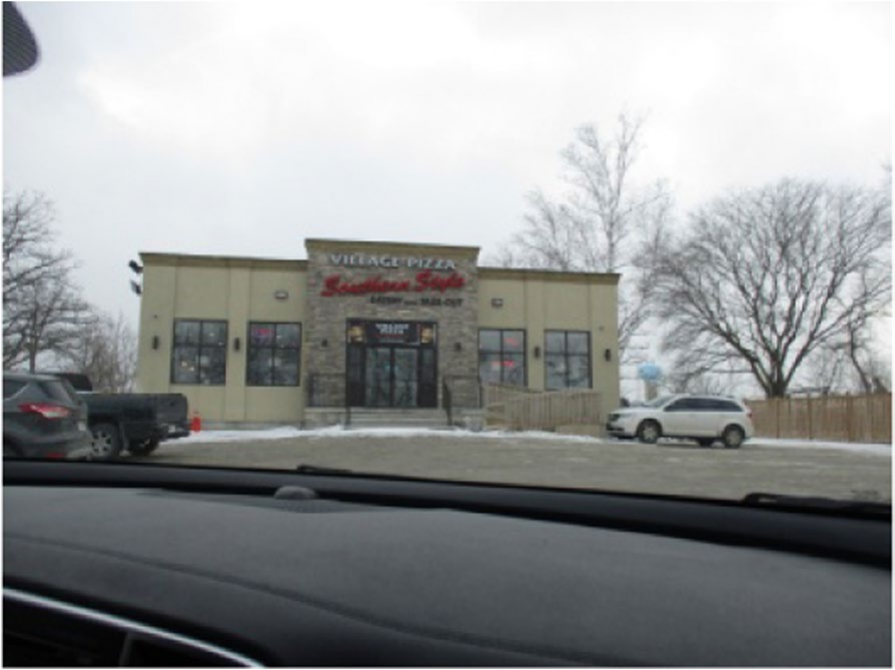
Popular community pizza restaurant (Maria, 15)

Participants described social norms and pressures related to food choices in the community. All but one participant spoke about a local pizza restaurant in their interviews, and two captured photos of it. Margaret recalled, *“we had Village one night, I didn’t take a picture of that… that’s like another go-to I think for people on the Rez.”* Maria also talked about the social aspects of the restaurant when discussing her photo (Fig. 7). She said, *“I feel like Village Pizza’s like a social thing especially if you’re picking up an order - it’s for your family or it’s for a party or something like that… or even if you’re going in to… sit down, like, dine-in, you’re usually with… other people too.”*

### Economic environment

As all participants were spending their own money on food, whether to eat out or help purchase groceries for their household, the topic of food expenses came up frequently during their interviews. Several spoke about how their personal finances combined with the local cost of food impacted their food choices. As highlighted earlier, a few of the youth reported that if they did not have money at school they would either get something from the Student Nutrition Program or miss lunch. Maria described how eating out depended on, “*if I have money sometimes I’ll go out with my friends and go out or just eat food from the* [cafeteria]*.”* Other participants indicated that cost was a barrier to purchasing food at school. Melody complained that some of the more nutritious cafeteria items such as fruits, salads, and wraps were not accessible to students due to their cost. She explained, *“we have salads there too* [in the cafeteria], *but those are pretty expensive.”*

**Fig. 8:**
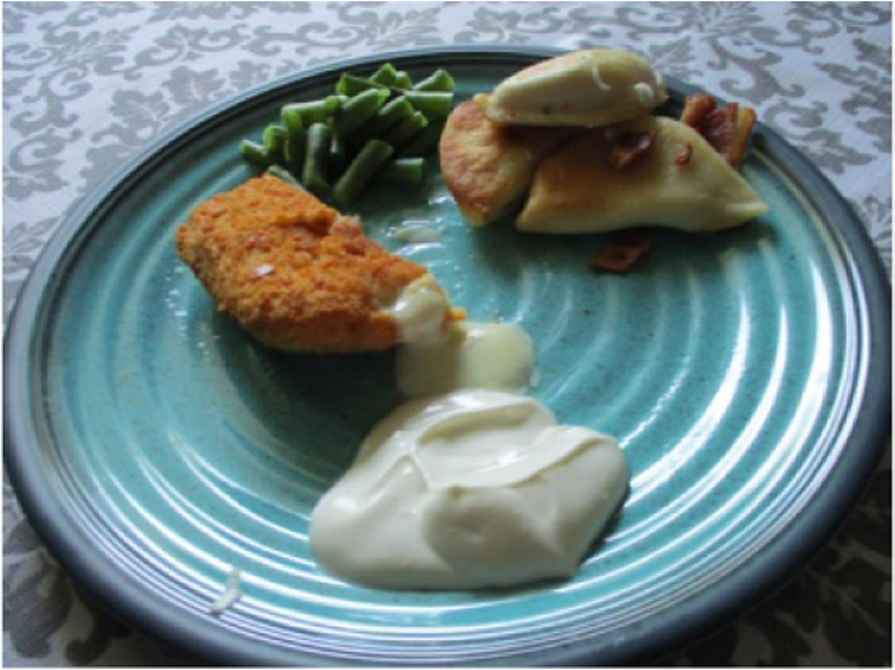
Meal of odds and ends (Margaret, 22)

Several women also talked about the economic environment on a broader scale that extended beyond their own food choices. Margaret spoke about how finances influenced the foods available in her household. The photo she took in Fig. 8 represented a meal shared with her family when funds were limited. As she described*, “that thing was gross…* [we had that] *‘cause there wasn’t anything left in the freezer. Usually when we have money for groceries we just eat the same things every week… until we run out and get creative… whatever’s in the cupboard.”* She also described how cost was a barrier to purchasing nutritious foods, stating, *“we’re poor so we can’t afford like super healthy food all the time.”* Georgia, who lives in the same home, explained how their family had made changes to their food habits to save money, stating *“recently we’ve been cooking food at home… because it’s cheaper”.*

**Fig. 9:**
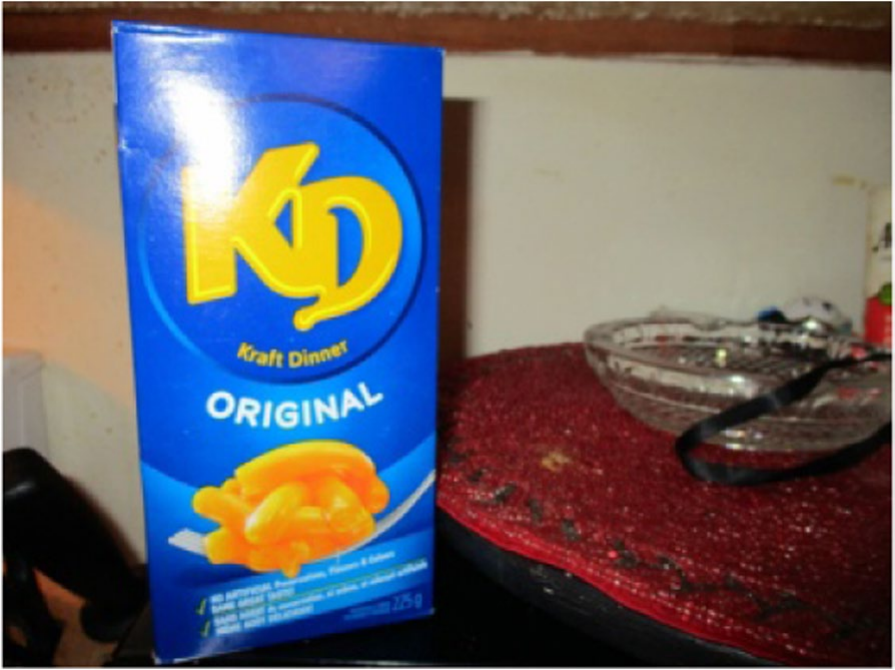
Affordable staple food (Margaret, 22)

Maria spoke more broadly about household food insecurity in the community*,* explaining that *“there’s people down here that don’t have the money for groceries sometimes so… breakfast, lunch, and dinner is for people who…got the money for groceries… like not even them… if they got kids too.”* Margaret suggested that cost was a barrier for other community members in accessing certain foods when she described her photo in Fig. 9. She said, “[people eat Kraft Dinner] *because it’s cheap…* [I have it] *‘cause I’m poor. It’s like a staple food around here… on the reserve anyway. I don’t know about Canadian families.”*

### Ecological environment

The youth’s photographs provided some context to discuss their food practices during the winter and summer seasons when the photos were taken. During their interviews they were also asked about any seasonal changes in their diets throughout the year. Four reported that their food intakes remained relatively consistent, regardless of season. Grace talked about the places her family accesses food as fairly static seasonally as, *“they don’t really change where they go.”* The only participant who noted seasonal variations in food intake was Melody who said: *“she* [my mom] *does a lot of… market shopping in the summertime… we definitely go to a grocery store more… in the wintertime.”* During the wintertime several participants discussed not shopping as regularly due to the cold weather. Melody said, *“I haven’t been really going to the store lately ‘cause it’s been really cold out so I try and just like stay inside.”*

**Fig. 10:**
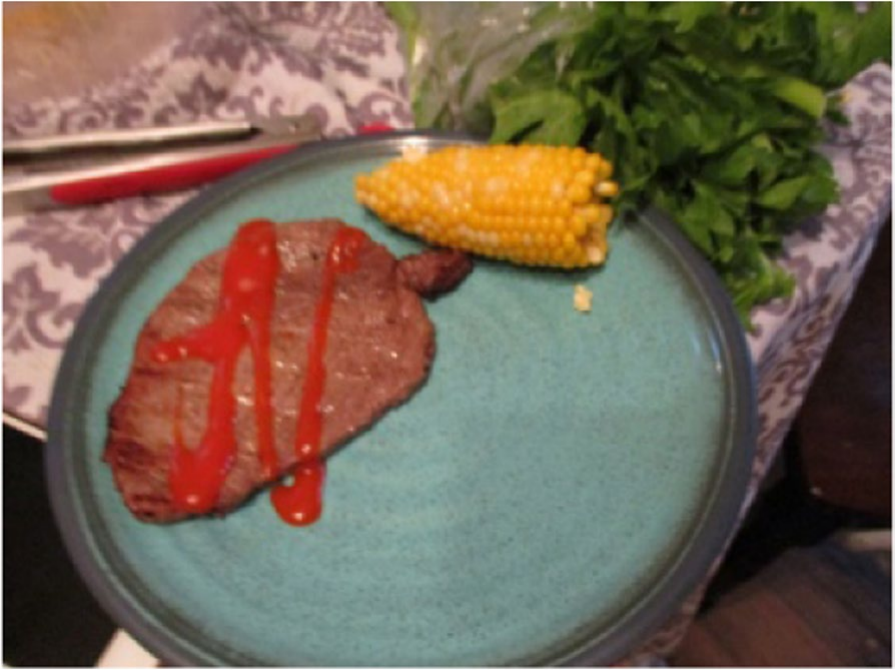
Seasonal summer corn (Georgia, 16)

While overall food choices did not appear to change drastically with the seasons, participants did talk about the local seasonal foods they most enjoyed and looked forward to each year. All but one of the youth mentioned eating freshly harvested corn in the summertime. Maria stated, *“I think the one thing that does change is corn… the summer is when we always have corn”*. Both Georgia and Margaret participated in the study during the summer. They both captured one or more photos that included corn (Fig. 10). Strawberries were another seasonal and locally available food that participants enjoyed. Margaret described seeking out strawberries at the local farmer’s market each year in addition to another local farm because, “*we just wanted strawberries since we didn’t go and pick them yet.”* Grace also spoke about an autumn family tradition to *“go apple picking with my family… apples just have… a lot of memories tied to them.”*

## Discussion

In this study, the built environment had a notable impact on food choice for the participating youth, most notably at home and in school settings. These locations represent the environments where youth spent the majority of their time and directly influenced participants’ diet quality, food preparation, and food acquisition behaviours. As all the youth lived at home with their families, most participants indicated their reliance on their parents or caregivers to purchase and prepare meals, which tended to positively impact diet quality and diversity. Genuis and colleagues [41] similarly found that Indigenous children were reliant on their parents for food provision. The group of Six Nations youth in this study also described how the transition from childhood to adolescence prompted increasing involvement in meal preparation and acquisition by their families, particularly for morning and mid-day meals when attending school. Evening meals served at home tended to be more nutritious compared to foods accessed outside of the home, and were the most consistently consumed meal by all participants. Other studies within Canada have reported on tendencies for youth to consume ultra-processed convenience foods at breakfast and lunch, while dinner often includes more homemade meals [50] that improve nutrient intakes and mealtime consistency [51].

Youth spend a significant amount of time at school, greatly impacting dietary behaviours as well [52]. Six Nations youth reported consuming primarily ultra-processed foods purchased from their school cafeteria or nearby fast-food establishments. This built environment may have increased the accessibility and availability of ultra-processed foods, as the proximity and number of restaurants close to schools tends to increase students’ likelihood of eating out [53] and diminishes diet quality [51]. Ultra-processed foods are affordable, accessible, and available to Indigenous communities in remote and urban settings [1, 54]. While the school setting can negatively impact students’ dietary quality through increased accessibility of ultra-processed foods, it also presents opportunities for positive influences [55–57], with several participants describing accessing fresh foods, such as fruit, from their School Nutrition Programs in the community.

Participants’ photographs consistently illustrated the linkages between these built environments and their social environment. During their interviews, family and friends were often mentioned in tandem when talking about meals at home and school. Family has been widely cited as a fundamental influence on the evolving food choices and preferences of youth [3, 52, 56, 58]. Family meals were described by all participants as both positive and negative experiences due to family and other social dynamics. Youth took photos and told stories of how food would bring friends and extended families together, highlighting how food practices are relational in nature. Many existing studies have focused on exploring how social relationships impact diet quality. However, the existing body of work does not capture the social benefits food can have beyond physical health. Participants in this study alluded to the role of food as part of their social and cultural wellbeing. Specific dishes and eateries were part of the social norms in their community. These findings align with the notion that food practices are inherently social as they reflect the political, economic, and cultural systems of place [52].

The social environments extended into the school settings in Six Nations. All four high school participants described lunch breaks as opportunities to socialize with their peers and eat together. Other research has indicated that for Indigenous female youth, socializing with peers often involves food, whereas male youth may be more likely to skip or rush meals in favour of other activities [52]. In the present study, youth similarly described how their peers influenced the types of foods they ate and, in some cases, their willingness to try new foods. Foods can hold social currency in peer groups, and youth are likely to make food choices to conform to social norms and gain peer approval [52, 59], which can include the ultra-processed foods that tend to be normalized [50].

Overlapping with the identified built and social environments, the youth also discussed the influence of the economic environment and how personal finances impact their ability to acquire food, as others studies have similarly found [52]. As youth become more responsible for their own food choices, ultra-processed foods become increasingly common because they are perceived to be more affordable options [50, 56, 58]. Participants described their desire to choose items that were both satisfying and affordable, often citing ultra-processed foods as meeting both of these needs. Financial constraints have been widely identified as a barrier to healthy eating [30, 58, 60]. Previous research has demonstrated that Indigenous communities within Canada experience disproportionate levels of economic challenges and food insecurity [12, 30, 60]. While participants in the present study did not directly discuss circumstances of household food insecurity, two participants described how food costs were a significant barrier to accessing food, particularly healthier food choices, for a number of families in the community.

Participants did not discuss impacts of the broader ecological environment, such as the sustainability of local food systems, on their everyday food choices, although some seasonal variations were discussed. The youth explained that their families primarily accessed groceries in a nearby city, which supports Joseph and colleagues’ [61] findings that Six Nations households tend to purchase foods off-reserve at larger grocery chains. A reliance on these market foods is likely associated with the limited seasonal change in food intake as the availability of foods in grocery stores is fairly consistent throughout the year. While participants did describe specific food items they would eat more often in the summer, such as fresh strawberries and corn, their overall dietary intake did not appear to be significantly impacted by their ecological environment compared to the built environment. In more rural and remote Indigenous communities where market foods are less available, the ecological environment has been described as a more important determinant of food choice [25, 26].

This study demonstrates that a complex collection of environmental factors converge to directly impact the individual food choices and practices of Haudenosaunee youth. All participants were eager to talk about their favourite foods, which encompassed ultra-processed foods and home-cooked dishes as well as traditional foods such as corn and strawberries. Other studies have also identified that taste and preference for foods is a strong indicator of their consumption among youth [41, 50, 56]. Taste is one of the top two influencers of food choice among youth, next to food availability [62]. Participants’ friends and families also directly influenced their food preferences and willingness to try new foods. Other researchers have identified how individual preference may not always influence food choice due to the presence of more important environmental factors such as physical as well as social environments. For example, Genuis and colleagues [41] found that, while children in their study described enjoying foods such as fruits, vegetables, and traditional foods, this did not always translate into their consumption due to the limited availability of these foods at home.

Several participants also described their perceptions of how their diet impacted their health feelings of wellness. While participants did share distinctions around healthy versus unhealthy foods, this knowledge did not appear to impact their food choices. Other studies have identified that while youth may have the intention and knowledge to eat healthy, this often does not translate into behaviour changes [56, 62]. Margaret was the only participant in this study who described her efforts to have more nutritious foods to be a role model for and promote the wellbeing of her child. Desjardins [50] also found that, in comparison to the younger adolescents in their study, young parents prepared more home-cooked meals as they were concerned with role modeling and teaching their children about healthy food choices.

While all study participants were responsible for making their own breakfast and lunch, the foods they described were mainly items requiring minimal preparation. Desjardins [50] similarly found that the consumption of ultra-processed foods was significantly higher among participating adolescents and that adolescents tend to have lower food literacy levels with improvements observed among young adults who were more competent and interested in improving their food skills. Aside from Margaret, the youth were almost exclusively dependent on family members to acquire and prepare food in their home environments. Lack of previous food skills education at home and school has previously been described as a barrier to acquiring food skills among Canadian university students [63]. Female youth in this study spoke about learning food skills at school, however, they did not refer to similar learning experiences at home even though several did express an interest to learn from family members.

## Conclusions and recommendations

Six Nations of the Grand River community members have expressed an interest in consuming more traditional foods and reconnecting to their Haudenosaunee foodways. The present study was, thus, developed to build on these community priorities while also addressing significant gaps in the literature. This study employed CBPR and Photovoice methodologies to explore Six Nations youth perceptions of traditional foods [31], their food environments, and determinants of food choice. Built, social, economic and ecological environments were each described by the youth as distinct yet inter-related determinants that converge to influence individual food choices among youth.

Of significance, this is the first study to explore the determinants of food choice among Indigenous youth and adds to the small body of literature that has explored Indigenous diets and food choice in a southern setting. As a methodology, a primary strength of using Photovoice is that it helps bring visibility to community-identified issues [37, 44]. Study participants had the autonomy to choose the subject and meaning of their photographs, which helped minimize researcher-participant dynamics while elevating and empowering the voices of youth [36, 37, 43]. Using Photovoice in this study allowed for the collection of rich and descriptive information regarding participants’ everyday and traditional food choices and experiences, shared through their photos and in their own words during individual interviews. The use of CBPR also strengthened this study. As a decolonizing methodology, CBPR engages community guidance of the research process to ensure studies benefit the communities involved [37, 38]. The vision for this study came from discussions with Six Nations Health Services staff and other community members. A local Research Assistant was hired from the community prior to beginning data collection. A Community Advisory was also formed and has met to share meals and make decisions regarding how this study has been implemented and how the results will continue to be shared. It was the intent in the development of the project that the findings will provide visual and social narratives to be considered in the development of relevant programming and resources within the community.

Overall, the study findings have generated important knowledge regarding Haudenosaunee female youth’s food environments and literacy. Several participants expressed a desire to enhance their food skills, which in some cases included learning how to prepare traditional dishes [31]. There may therefore be a need for more food literacy education opportunities for youth, emphasizing hands-on food skills and other learning opportunities from Knowledge Keepers and Elders in the community. Two participants described how the majority of their traditional food experiences and knowledge came from attending a traditional elementary school [31]. This privately run school in the community offers language immersion programs and unique curriculums to preserve local knowledges, while preparing students to pursue further education or opportunities in the workforce. These examples demonstrate how schools offer an ideal setting for young people to acquire food skills and share knowledge. As such, it would be worthwhile to explore how other schools in the community could better integrate food literacy programming and traditional food education into their curriculums. The incorporation of this learning into school curricula would also address the identified barrier of time constraints among female youth, through reducing their need to seek learning opportunities on their own time outside of the school environment.

The social environments the youth documented and described in this study are also central to consider. Study participants shared how their families directly influenced their daily meal and consumption patterns, while peer relationships impacted their food choices outside of the home. Youth described food traditions they had experienced with loved ones, demonstrating how food encourages them to connect inter-generationally. Providing food skills programming in various social settings is an avenue to consider. In particular, it would be worth exploring opportunities to offer food skill activities within existing youth programs in the community, as these provide an ideal social setting for peer learning. Another option would be to further expand the existing adult and Elder food programming offered by Six Nations Health Services to engage more youth as participants and further encourage inter-generational knowledge transfer.

As participants described a level of dependence on their family members to acquire and procure foods at home, the engagement of family must also be taken into account. While participants talked about and illustrated the positive influences of home environments on dietary quality and diversity, there was no mention about learning food skills in their homes. This important finding suggests that engaging both parents/caregivers and youth in family–based education and programming would be important as has also been suggested previously [3, 31] as an important determinant of increased traditional food consumption [31]. The majority of participating female youth accessed traditional foods at community events and described how these foods had an important role in their community. These previously published findings suggest that it would be of benefit to expand existing programming that connects community members through food throughout the lifecycle.

Additional barriers to healthy food choices, food skills, and traditional food consumption such as economic constraints impacting individual food choices must also be considered in the development of relevant programming and resources for Haudenosaunee youth. The planning and delivery of food skills education activities in schools and the community should be informed and, when possible, led by youth to help address their unique food environments, while enhancing peer interest and engagement through their empowerment as leaders. Economic constraints could also be incorporated in food skills programming through highlighting lower cost, locally grown and sourced foods. Policies aimed to enhance the affordability and accessibility of nutritious foods in school environments would be impactful. Broader community-identified solutions to reduce systemic circumstances of chronic food insecurity would be required to address these issues identified by participants as a significant barrier to sustainable food consumption.

Recognizing the limitations of the small sample of participants, it is also recommended the next stages of this ongoing research should prioritize the recruitment of participants of diverse ages and genders. Engaging more youth in this study would add to the strength of these findings and provide more informed recommendations to the community. It is also critical that the Community Advisory Group continue to guide how the broader research project unfolds, including future plans for disseminating these findings to the community at-large and beyond. This study serves as a unique example of how to explore the traditional and everyday food experiences of Indigenous youth and recommendations will inform the development of new as well as existing initiatives and resources to enhance the holistic wellbeing of youth and the broader community of Six Nations. Methodologies and methods could be adapted and utilized to explore the determinants of food choice among Indigenous youth in other environmental settings, adding to the small but growing body of literature on this important subject. Such research could inform broader policy solutions – relevant to school food programs or supports for lowerincome families – that are required to address the more distal determinants of food choice touched on by the female youth who visually captured their food environments and shared their stories.

## Data Availability

The datasets used and/or analysed during the current study are available from the corresponding author on reasonable request.

## Footnotes

1 The definition of “traditional” varies throughout the literature. In this article, we use the term to indicate foods that are available to an Indigenous Peoples from the local natural environment, including both plant and animal species [9, 17].

2 Pre-contact foods denotes foods that were available and consumed pre-settler contact [4].
